# Intelligent Fall-Risk Assessment Based on Gait Stability and Symmetry Among Older Adults Using Tri-Axial Accelerometry

**DOI:** 10.3389/fbioe.2022.887269

**Published:** 2022-05-13

**Authors:** Wei-Chih Lien, Congo Tak-Shing Ching, Zheng-Wei Lai, Hui-Min David Wang, Jhih-Siang Lin, Yen-Chang Huang, Feng-Huei Lin, Wen-Fong Wang

**Affiliations:** ^1^ Department of Physical Medicine and Rehabilitation, National Cheng Kung University Hospital, College of Medicine, National Cheng Kung University, Tainan, Taiwan; ^2^ Department of Physical Medicine and Rehabilitation, College of Medicine, National Cheng Kung University, Tainan, Taiwan; ^3^ Ph.D. Program in Tissue Engineering and Regenerative Medicine, National Chung Hsing University, Taichung, Taiwan; ^4^ Graduate Institute of Biomedical Engineering, National Chung Hsing University, Taichung, Taiwan; ^5^ Department of Computer Science and Information Engineering, National Yunlin University of Science and Technology, Yunlin, Taiwan; ^6^ Institute of Biomedical Engineering, College of Medicine and College of Engineering, National Taiwan University, Taipei, Taiwan; ^7^ Institute of Biomedical Engineering and Nano-medicine, National Health Research Institutes, Zhunan, Miaoli, Taiwan

**Keywords:** fall risk, gait, stability, symmetry, older adults, accelerometry

## Abstract

This study aimed to use the k-nearest neighbor (kNN) algorithm, which combines gait stability and symmetry derived from a normalized cross-correlation (*NCC*) analysis of acceleration signals from the bilateral ankles of older adults, to assess fall risk. Fifteen non-fallers and 12 recurrent fallers without clinically significant musculoskeletal and neurological diseases participated in the study. Sex, body mass index, previous falls, and the results of the 10 m walking test (10 MWT) were recorded. The acceleration of the five gait cycles from the midsection of each 10 MWT was used to calculate the unilateral *NCC* coefficients for gait stability and bilateral *NCC* coefficients for gait symmetry, and then kNN was applied for classifying non-fallers and recurrent fallers. The duration of the 10 MWT was longer among recurrent fallers than it was among non-fallers (*p* < 0.05). Since the gait signals were acquired from tri-axial accelerometry, the kNN F1 scores with the x-axis components were 92% for non-fallers and 89% for recurrent fallers, and the root sum of squares (RSS) of the signals was 95% for non-fallers and 94% for recurrent fallers. The kNN classification on gait stability and symmetry revealed good accuracy in terms of distinguishing non-fallers and recurrent fallers. Specifically, it was concluded that the RSS-based *NCC* coefficients can serve as effective gait features to assess the risk of falls.

## Introduction

Falls are a major public health problem in adults aged >65 years worldwide. Approximately 30% of adults who have experienced a fall once tend to fall each year, and medically injurious falls occur in 33% of older fallers (Shumway-Cook et al., 2009). Falls are identified as one of the leading causes of injury-related mortality among older adults, and a reduction in mobility, disability, and multiple comorbidities tends to occur. Beyond individual impairment, falls and fall-related injuries place great financial and healthcare burdens on the families of the fallers and their communities. Because fall incidence rates increase in parallel with the continually increasing aging population globally, the fall-related impact on social and economic aspects is expected to become more profound. Therefore, early detection of older adults in high risk of falls is important for preventing falls and reducing their incidence.

Several factors have been demonstrated to increase fall risk in older adults, including muscle weakness, gait abnormalities, neurological deficits, visual and hearing impairments, orthostatic hypotension, and cognitive problems (Ambrose et al., 2013). Gait abnormalities have been consistently recognized as a potential risk factor for falls (Tinetti et al., 1988). The gait of older adults is less coordinated with higher variability than that of younger adults (Herman et al., 2005). However, in a previous study, age-associated differences were not detected in gait variability, even though older adults walked slower than younger adults (Hausdorff et al., 1997).

Due to the latest developments in wearable technology, instrumental gait analyses, that is, gait analyses using inertial measurement units (IMUs), have considerable benefits over traditional methods, including the fact that they are noninvasive and highly accurate, and the technology has a space-saving design (Saremi et al., 2006). By processing data from wearable sensors to assess the risk of falls, several studies adopted various machine learning algorithms with an accuracy that ranged from 0.77 to 0.92 (Drover et al., 2017; Hua et al., 2018; Tunca et al., 2020; Wu et al., 2020). Other studies have utilized conventional statistical procedures to differentiate between non-fallers and fallers cross-sectionally or longitudinally (Caby et al., 2011; Cella et al., 2020). Hence, regarding the heterogeneity of the studies, including the data acquisition, processing, application, and study design, no consensus has been reached on the gait parameters that are most appropriate to use to study falls, especially in older adults (Sun and Sosnoff, 2018).

Gait symmetry and stability are two important aspects of instrumental gait analysis. Gait symmetry is related to the signal similarity between the bilateral lower limbs, whereas gait stability is related to the signal similarities among consecutive gait cycles. In fact, the walking process is composed of a series of interdependent gait cycles, where each gait cycle has its own individual and specific structure embedded in the IMU gait data. Therefore, the processing of IMU data during walking tasks makes the evaluation of the degree of gait symmetry (Gouwanda and Senanayake, 2011; Lien et al., 2017) and stability (Kobsar et al., 2014) possible. In addition, the IMU gait data processed by using a normalized cross-correlation (NCC) analysis can also be used to improve the results of intelligent recognition of fall risks. NCC analyses have recently been utilized to study the degree of bilateral gait symmetry in legs in Parkinson’s disease (Park et al., 2012) and to differentiate fallers from non-fallers among patients who have experienced a stroke (Lien et al., 2017). Actually, features derived from NCC analyses can highlight gait stability and symmetry among different gait signals and also reveal valuable information about the mobility of patients (Ogihara et al., 2020). To intelligently assess fall risk, the use of the k-nearest neighbor (kNN) algorithm combined with NCC analysis to inertial measurement gait signals was proposed in this study. Due to the large differences in the walking styles of different individuals or subjects, the inertial measurement gait signals in accelerometry obtained from different individuals also have significant variations. When these gait signals are used to calculate gait symmetry and stability, a large number of extreme values will interfere with the accuracy of the classification results. In this case, the use of an NCC analysis can exclude the extreme conditions of the measured gait signal, which is conducive to the improvement of the derived gait symmetry and stability features of gait symmetry and stability. In addition, kNN is a non-parametric classification method that does not make any assumptions about the underlying data distribution. In this way, the classification accuracy of kNN can be significantly improved.

To the best of our knowledge, no study has used NCC analysis data combined with kNN algorithms to distinguish between older non-fallers and recurrent fallers. Therefore, in this study, we employed a kNN algorithm, which combines the gait stability and symmetry features derived from an NCC analysis, to develop a novel fall-risk assessment model in older adults.

## Materials and Methods

### Experiment Configuration and Instrumentation

**FIGURE 1:**
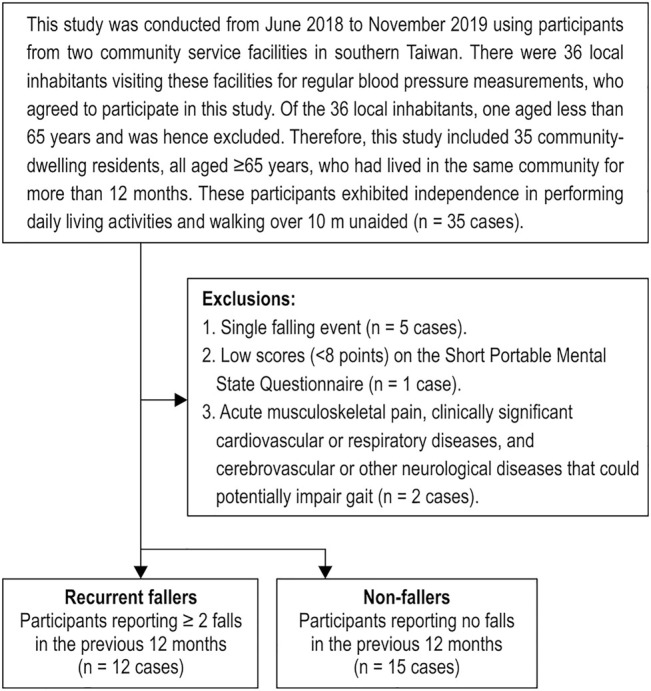
Flow chart illustrating the inclusion and exclusion criteria of the study participants.

This cross-sectional study was conducted from June 2018 to November 2019 enrolling participants from two community service facilities in southern Taiwan. There were 36 local inhabitants that visited these facilities for regular blood pressure measurements during the study period, and that agreed to participate in this study. Of the 36 total local inhabitants, one was less than 65 years old and hence excluded from this study. Ultimately, this study included 35 community-dwelling residents who had visited these facilities for regular blood pressure measurements, all aged ≥65 years, who had lived in the same community for more than 12 months. These participants exhibited independence in performing daily living activities and walking over 10 m unaided. The following exclusion criteria were then applied to the group: single faller (*n* = 5 cases), those with low scores (<8 points) on the Short Portable Mental State Questionnaire (Lien et al., 2015) (*n* = 1 case), or those with acute musculoskeletal pain, clinically significant cardiovascular or respiratory diseases, or cerebrovascular or other neurological diseases that potentially impaired gait (*n* = 2 cases). After exclusion, 27 adults aged ≥65 years were recruited (Figure 1). This study was approved by the Institutional Review Board of the National Cheng Kung University Hospital (approval no. A-ER-105-393) and conformed to the Declaration of Helsinki. All participants provided written informed consent before entering the study.

**FIGURE 2:**
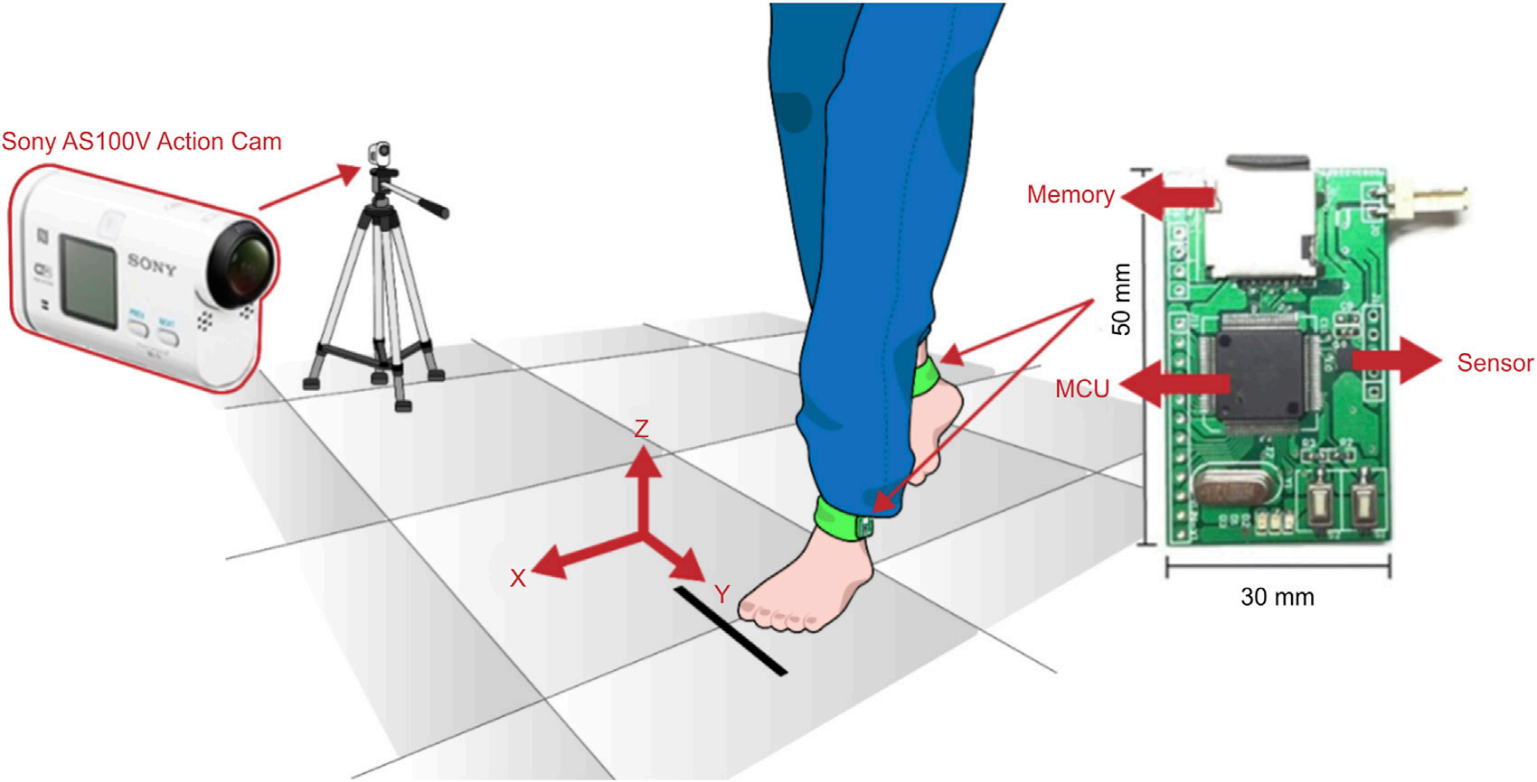
Wearable sensors, measuring coordinates, and gait image records for the experiment configuration. MCU: microcontroller unit.

Face-to-face interviews were conducted to record sex, age, body mass index (BMI), and fall history in the previous 12 months. A stopwatch was used to measure the timed 10 m walk test (10 MWT), which required the participant to walk twice on a 10 m walkway at a self-selected walking speed wearing bilateral ankle accelerometers (Figure 2). The average time(s) from the two 10MWTs were calculated.

A tri-axial accelerometer (LIS3DH; STMicroelectronics, Geneva, Switzerland) at a ± 16-*g* full scale was applied 3 cm above the lateral malleolus on each lateral ankle using custom-made bulk straps (Figure 2) (Wang et al., 2018) to quantify gait acceleration along the x (anterior-posterior), y (medio-lateral), and z (vertical) axes at a sampling rate of 120 Hz. Simultaneously, gait images were recorded with an action camera (Sony AS100V) to assist in verifying the details of the gait. The acceleration signals of the five midsection gait cycles were inputted into MATLAB for later processing.

### Data Acquisition and Processing

**FIGURE 3:**
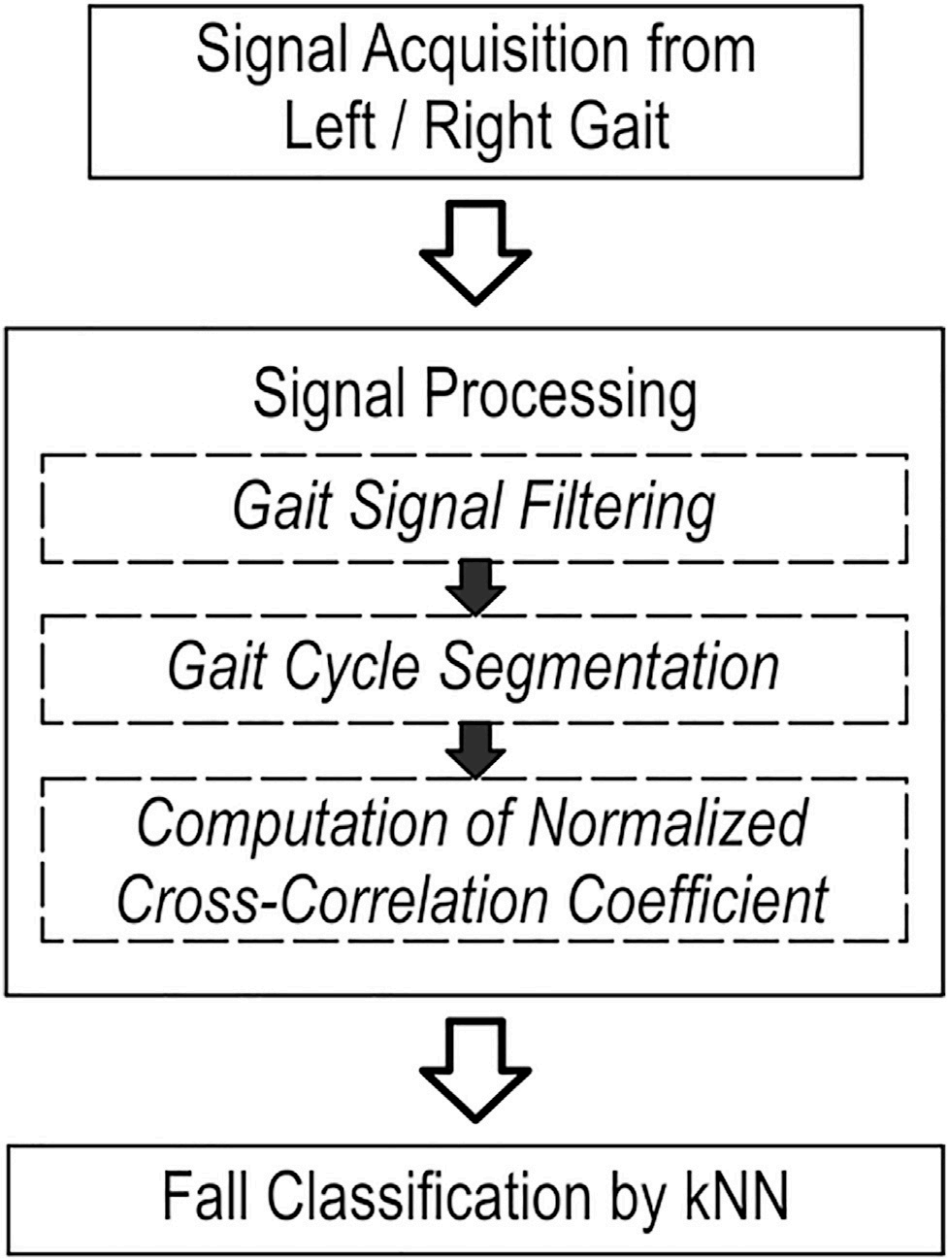
Working procedure for gait signal data acquisition, processing, and classification. kNN, k-nearest neighbor.

**FIGURE 4:**
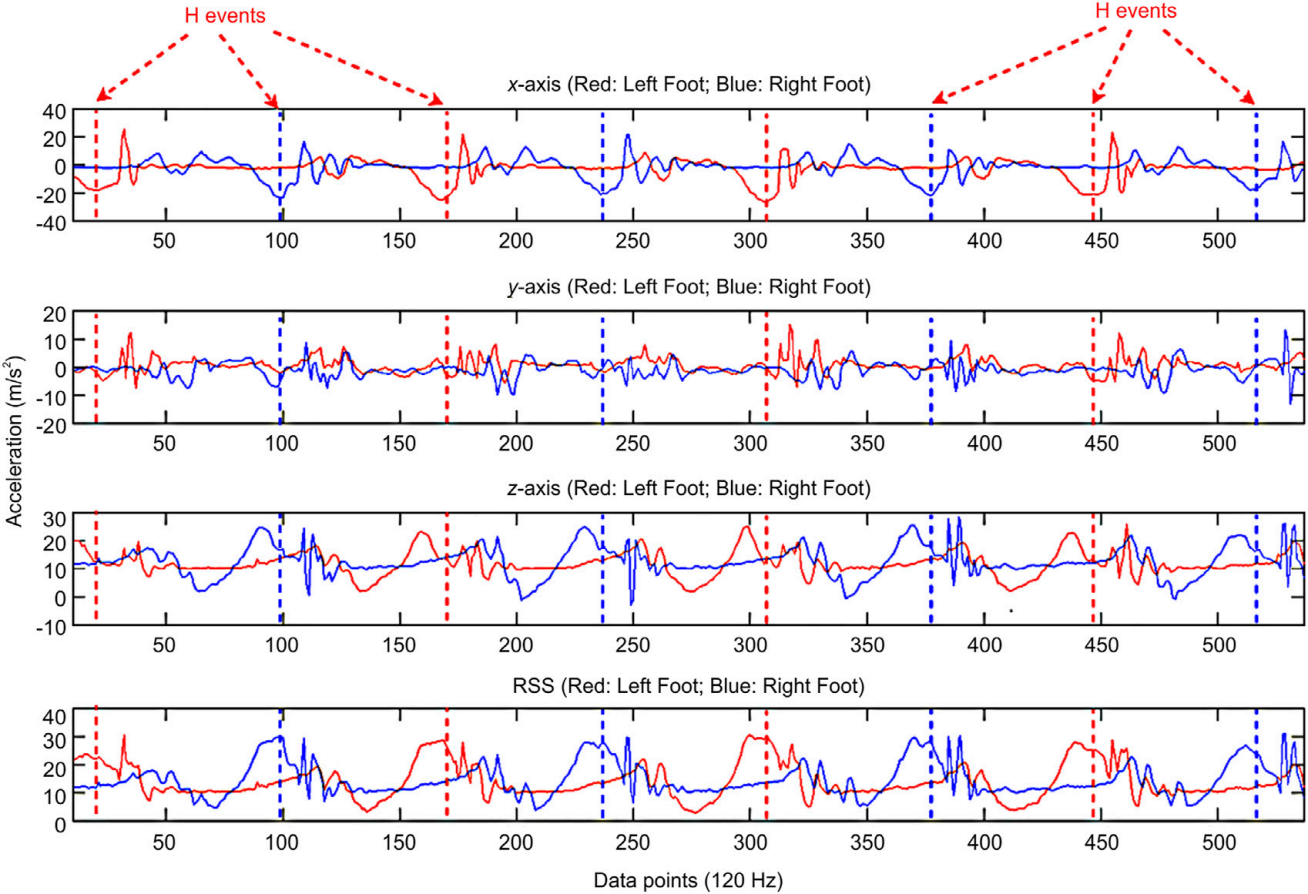
Gait signal data after filtering and segmentation. H, heel strike; RSS, root sum of squares.

The gait signal processing flowchart depicted in Figure 3 was used to process the acquired gait data from the participants. After raw acceleration signals were acquired from the left/right legs, the signals were filtered using an eighth order low-pass Butterworth filter. The cut-off frequency was set to 20 Hz (Wang et al., 2018). The filtered gait acceleration signals are shown in Figure 4. In the gait acquisition experiment, we applied a strategy to synchronize the accelerometers and action camera to achieve a sampling rate of 120 Hz, thereby facilitating the subsequent signal-processing stages (Wang et al., 2018). By observing the recorded gait images, we found that the heel strike events in Figure 4, the heel strike actions in the gait cycles, were obvious and could be easily delineated from the valley accelerations of the ankle along the x-axis (Lien et al., 2017). Therefore, the heel strike event was used to segment the IMU gait data into consecutive gait cycles (Lien et al., 2017).

### Normalized Cross-Correlation Analysis

A three-dimensional (3D) acceleration vector (a_Rx_, a_Ry_, a_Rz_) was used to represent the magnitudes of one sampled signal from the right ankle tri-axial accelerometer and vector (a_Lx_, a_Ly_, a_Lz_) from the left ankle, respectively (Lien et al., 2017). At the same time, the root sum of the squares (RSS) of the left/right acceleration vectors was calculated using Eqs 1, 2, respectively.

$$\mathrm{RS}S_{R}=\sqrt{a_{\mathrm{Rx}}^{2}+a_{\mathrm{Ry}}^{2}+a_{\mathrm{Rz}}^{2}}$$ (1)

$$\mathrm{RS}S_{L}=\sqrt{a_{\mathrm{Lx}}^{2}+a_{\mathrm{Ly}}^{2}+a_{\mathrm{Lz}}^{2}}$$ (2)

Among the older adults, an evaluation scheme was proposed based on an NCC analysis to investigate fall risk. The evaluation scheme focused on the properties of *gait stability* on the same side, i.e., gait comparison of the left or right side, and *gait symmetry* on both sides, i.e., gait comparison of left over right or right over left. For the cross-correlation analysis, *α* and *β* were two discretely sampled gait signals, where the sampled sizes were *N* and *M* (*M* ≥ *N*), respectively. The normalized cross-correlation function (*NCC*_*α,β*_(*t*)) is given as follows (Lien et al., 2017):

$$\mathrm{NC}C_{\alpha ,\beta }\left(t\right)=\frac{\sum_{i=1}^{N}\left(\alpha_{i}-\bar{\alpha }\right)\left(\beta_{i+t}-{\bar{\beta }}_{t}\right)}{\sqrt{\sum_{i=1}^{N}{\left(\alpha_{i}-\bar{\alpha }\right)}^{2}\sum_{i=1}^{N}{\left(\beta_{i+t}-{\bar{\beta }}_{t}\right)}^{2}}}$$ (3)

where *t* is a shift parameter ranging from 0 to M*–*N, and $\bar{\alpha }$ and $\bar{\beta_{t}}$ are the means of the *α* signal series (*α*_1_,…, *α*_*N*_) and *β* signal partial series (*β*_*t*+1_,.., *β*_*t*+*N*_), respectively. Note that, for each value of *NCC*_*α*,*β*_(*t*), the correlation coefficient varied between −1 and 1.

**FIGURE 5:**
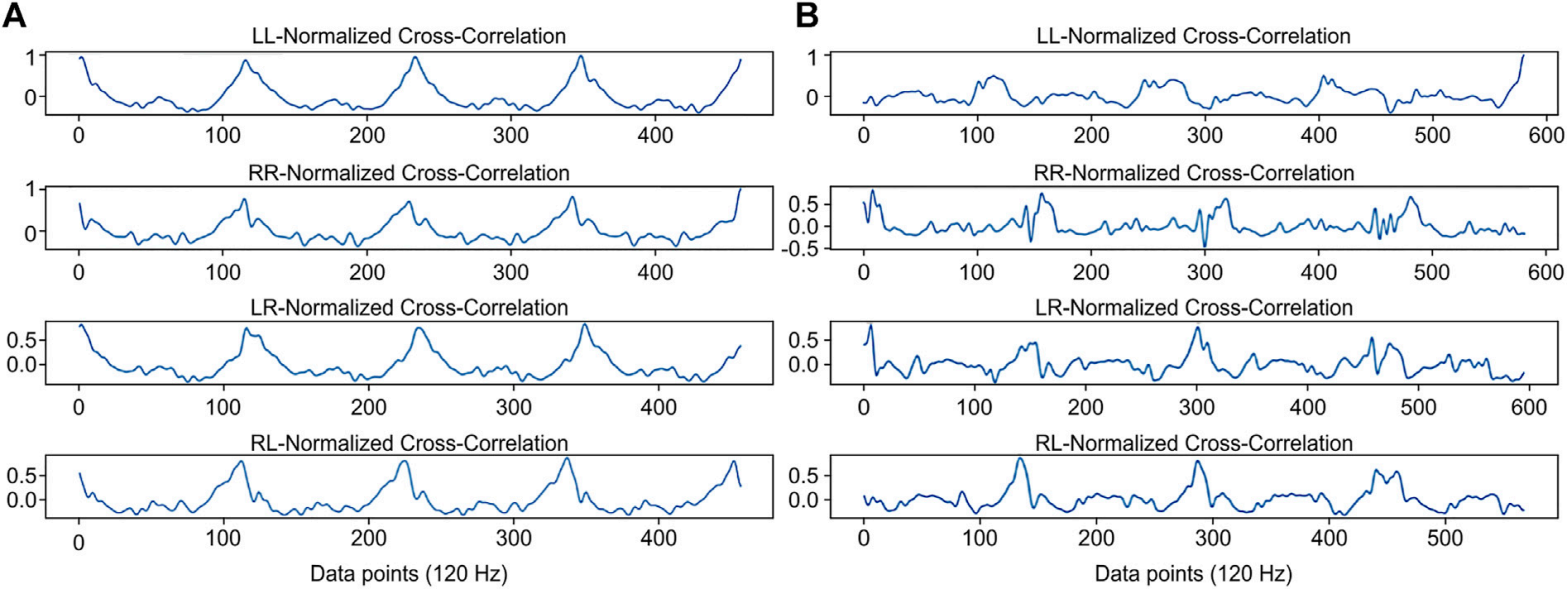
The normalized cross-correlation coefficients (*NCC*) in left-to-left *NCC* series (LL), left-to-right *NCC* series (LR), right-to-right *NCC* series (RR), and right-to-left *NCC* series (RL) for **(A)** non-fallers and **(B)** recurrent fallers.

To explore gait stability and symmetry, the five middle strides from the gait data obtained from each walking trial were used. By successive computation of *NCC*_*α,β*_ *(t)* using Eq. 3 for *t* ranging from 0 to (*M*–*N*), the produced *NCC* series was generated to reveal the temporal locations of abnormal gait in the acceleration gait signals using a sliding window of the *β* signal partial series to the α signal series. To compute *NCC*_*α,β*_ *(t)*, *t* = 0..(*M*-*N*), a one-gait signal record was obtained from one walking trial and was labeled as *β.* Then, the signals of one complete gait cycle were picked up from the chosen gait signal record of the same side or a gait signal record of the other side in the same walking trial, which was labeled *α*. Consequently, four *NCC* series were generated according to the combinations of the left/right sides and *α*/*β* sequences in *NCC*_*α,β*_ *(t)*, which were denoted as follows: left-to-left *NCC* series = LL = {*NCC*_*Lα*,*Lβ*_(*t*) |*t* = 0..(*M*-*N*)}, right-to-right *NCC* series = RR = {*NCC*_*Rα*,*Rβ*_(*t*)|*t* = 0..(*M*-*N*)} for the exploration of gait stability, left-to-right *NCC* series = LR = {*NCC*_*Lα*,*Rβ*_(*t*) | *t* = 0..(*M*-*N*)}, and right-to-left *NCC* series = RL = {*NCC*_*Rα*,*Lβ*_(*t*)|*t* = 0..(*M*-*N*)} for the exploration of gait symmetry. These *NCC* series were considered in the classification of recurrent fallers and non-fallers using the kNN algorithm. Figure 5 shows the LL, LR, RR, and RL for one non-faller and one recurrent faller.

### Fall Classification

The variations in the attached orientation and position of the wearable sensors in each phase of the gait signal acquisition affected the accuracy of the acquired signal. Notably, four types of IMU gait data sources (*i.e.*, x-/y-/z- axis components and RSS values) derived from the acquired acceleration gait signals could be employed to generate the *NCC* series for the fall classification. To cope with this issue, application of the RSS values was necessary to remove the sensing directionality because all the x-/y-/z- axis components, which are direction-related, were transformed to a directionless magnitude––RSS.

To utilize the kNN algorithm, we proposed two parametric functions, *mean* (M) and *variability* (V), to construct feature vectors for the classes of recurrent fallers and non-fallers. To calculate the M of LL, for example*,* M (LL), only the arithmetic mean of the set LL was calculated. The same operation could be applied to the M of RR, LR, and RL. V could be measured using Eq. 4 as follows:

$$V\left(\mathrm{NCC}\_\mathrm{set}\right)=\frac{\max\left(\mathrm{NCC}\_\mathrm{set}\right)-\min\left(\mathrm{NCC}\_\mathrm{set}\right)}{\mathrm{mean}\left(\mathrm{NCC}\_\mathrm{set}\right)}$$ (4)

where the *NCC_set* can be one of the LL, RR, LR, and RL sets, and where max (*NCC_set*), min (*NCC_set*), and *mean* (*NCC_set*) provide the maximum, minimum, and average values of the *NCC_set*, respectively. Similarly, the *NCC_set* selection for computing Eq. 4 could be one of the LL, RR, LR, and RL sets.

To identify the gait patterns of recurrent fallers or non-fallers using the kNN algorithm, a feature vector was defined as follows:

Gait_Pattern=(M(RR), V(RR), M(LL), V(LL), M(RL), V(RL),M(LR), V(LR), gait_label ) (5)

where *gait_label* was set at 0 for the purpose of representing the class of recurrent fallers and was set at 1 for non-fallers. Remarkably, all components in the *Gait_Pattern* vector, with the exception of *gait_label*, could be derived from the IMU gait data sources. In the kNN algorithm, a vector as the one mentioned above was classified by a plurality vote of its nearby vectors, with the object being designated to the category most common among its k-nearest neighbors quantified using the Euclidean distance, such as ║*Gait_Pattern*║. In addition, another unresolved issue in kNN is the optimum choice of the K value. After a series of 5-fold cross-validation trials for K, the choice of K values was based on the IMU gait data sources from the x-axis component of the 3D acceleration signals and RSS values up to a feasible extent based on the error rate.

### Statistical Analyses

The clinical characteristics of community-dwelling older adult non-fallers and recurrent fallers were compared. According to Caby et al. (2011), for 95% power at a 5% two-tailed significance level, we required complete data for at least 16 cases (eight cases in each study group) to detect the difference in the wearable sensor gait feature extraction, with power analysis using G*Power 3.1 software (Univesitat Kiel, Germany) (Faul et al., 2007). Descriptive statistics were calculated as the number of units, percent, mean ± standard deviation, and median (interquartile range) values. Distributions of the continuous variables were evaluated using the Shapiro–Wilk test. According to the results of the normality tests, a two-sample t-test or Mann–Whitney U test was used to make between-group comparisons of the numerical variables. In the case of the categorical variables, because more than 20% of cells had expected frequencies <5, we used Fisher’s exact test. The performance of the kNN algorithm is described in the Supplementary Methods. Statistical significance was set at *p <* 0.05. All tests were two-tailed. All statistical analyses were performed using the SAS statistical package (version 9.3; SAS Institute, Cary, NC, United States).

## Results

To assess fall risk in an older population, 12 older adults who had fallen at least twice within 1 year and 15 older adults without falling experience were recruited. The average age of the 15 older non-fallers was 80.7 ± 4.3 years (range 75–89 years, 4 men). The average age of the 12 older recurrent fallers was 78.8 ± 3.2 years (range 74–84 years, 4 men). The 10 MWT score was higher in the recurrent fallers than in the non-fallers (*p* < 0.01). The recurrent fallers had a longer stance phase and a shorter swing phase than the non-fallers during the gait cycle (*p* < 0.05). No group differences were found in terms of sex, age, BMI, and the stance phase (as a percentage) or the swing phase of the gait cycle (Table 1).

**TABLE 1:** Basic characteristics of the old non-fallers and recurrent fallers.

Variables	Non-fallers, *n* = 15	Recurrent fallers, *n* = 12	*p* value
Age, years	80.7 ± 4.3	78.8 ± 3.2	0.2 (n.s.)
Sex, female (%)	11 (73.3)	9 (75)	1.0 (n.s.)
BMI, kg/m^2^	23.2 (20.6–24.2)	23.6 (22.2–24.7)	0.3 (n.s.)
10-m walk test, s	8.43 (8.40–8.49)	8.80 (8.63–9.19)	<0.01**
Stance of gait cycle (%)	57.4 ± 2.2	58.3 ± 4.9	0.53 (n.s.)
Swing of gait cycle (%)	42.6 ± 2.2	41.7 ± 4.9	0.11 (n.s.)

Values are n (%), mean ± SD, or median (interquartile range). BMI, body mass index; n.s.: not statistically significant. *p < 0.05; **p < 0.01.

To assess the risk of falls in the older adults, the two prospective observations of gait stability and symmetry described above were conducted and tested. To explore the significance of gait stability and symmetry in the classification of recurrent fallers and non-fallers, Figure 5A shows that non-fallers had higher maximal values and smoother gait patterns during stability transitions on the left and right legs than the recurrent fallers (Figure 5B). In terms of gait symmetry, Figure 5A shows that non-fallers had higher maximal values and a smoother gait pattern during symmetry transitions in the left-over-right or right-over-left gait comparisons than the recurrent fallers (Figure 5B). However, the comparison in the same situations shown in Figure 5B revealed the opposite for a faller adult. That is, recurrent fallers had poor gait stability and symmetry on both sides. Therefore, gait stability and symmetry appear to be excellent characteristics for classifying older adults as recurrent fallers or non-fallers.

**FIGURE 6:**
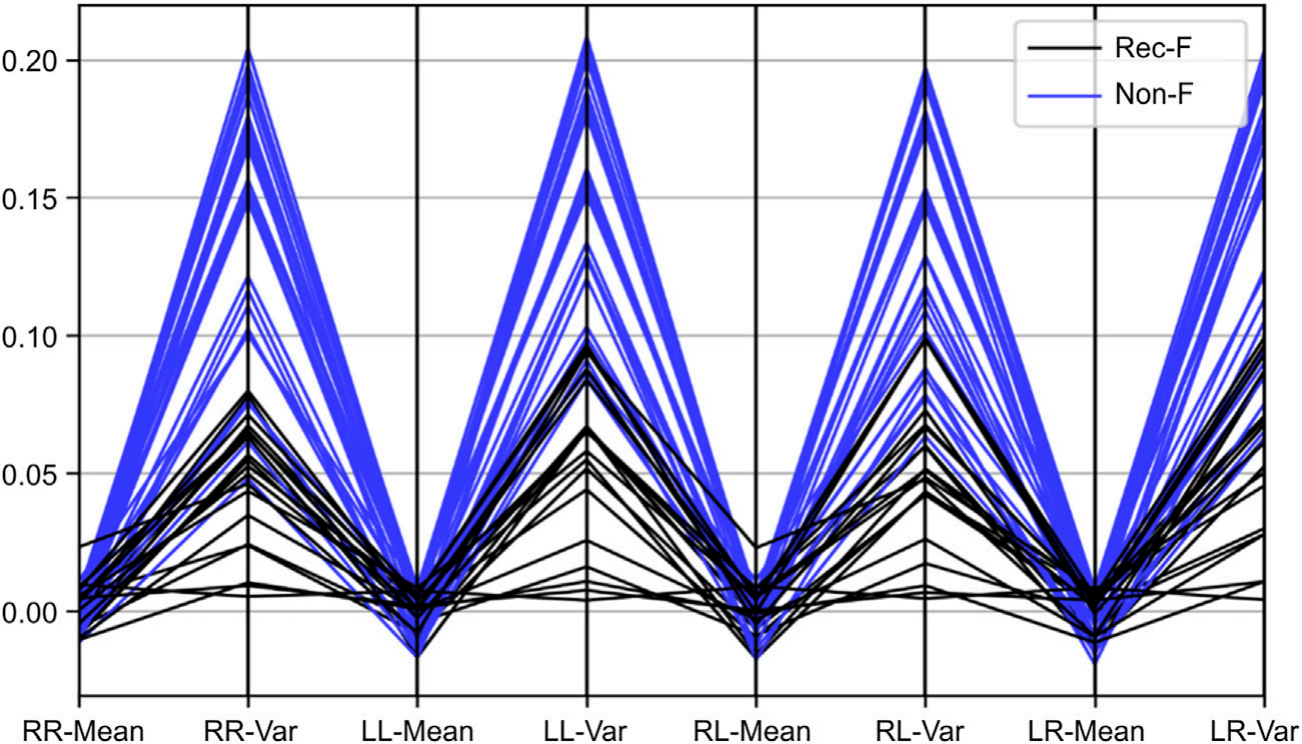
The distribution of the values of mean (Mean) and variability (Var) of left-to-left *NCC* series (LL), left-to-right *NCC* series (LR), right-to-right *NCC* series (RR), and right-to-left *NCC* series (RL) for recurrent fallers (Rec-F) and non-fallers (Non-F) using a line chart.

**FIGURE 7:**
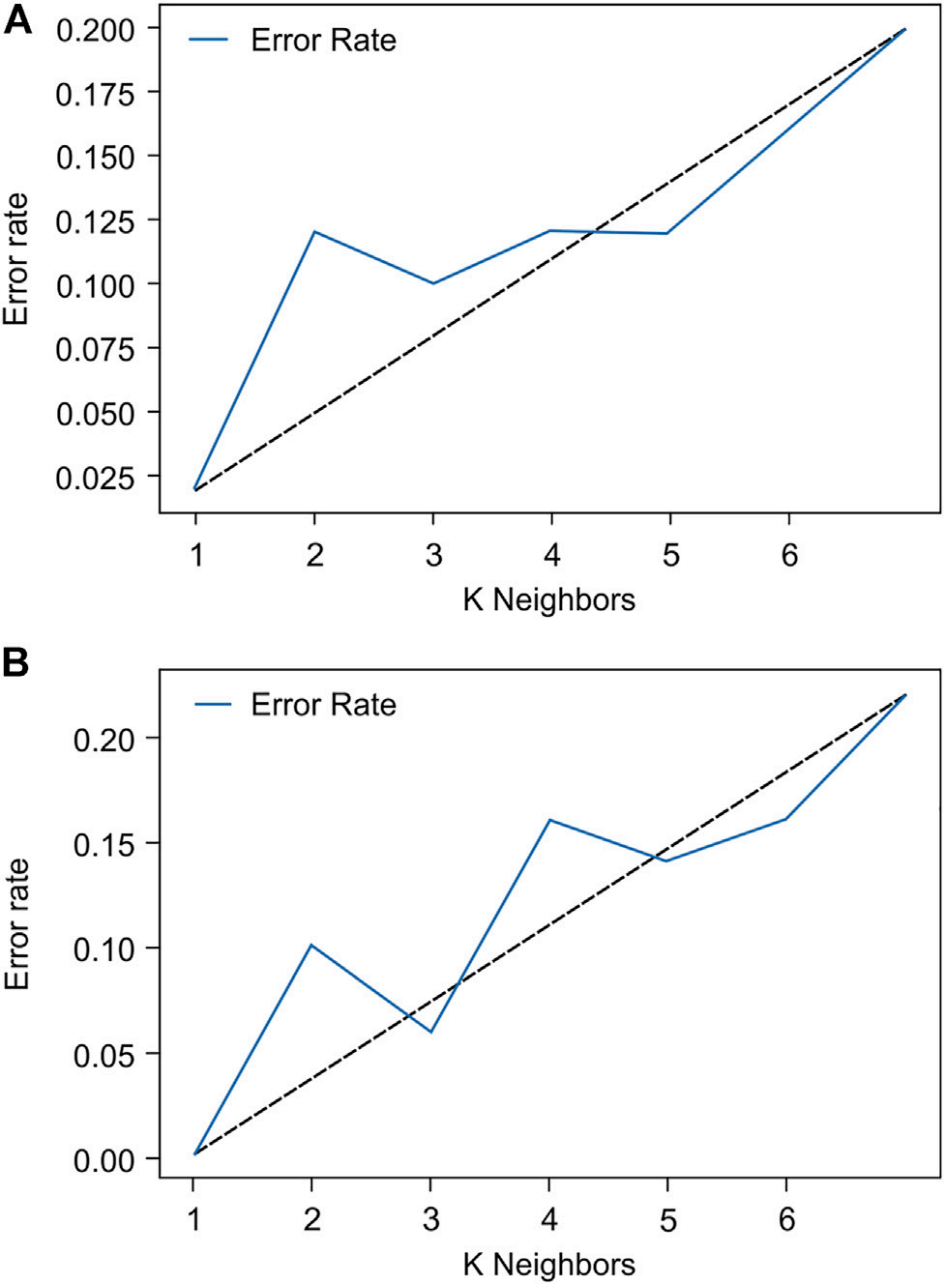
The choice of K neighbors for the k-nearest neighbor with **(A)** the x-axis component of the three-dimensional acceleration signals and **(B)** the root sum of squares values.

The distribution of the values of mean and variability of LL, LR, RR, and RL for recurrent fallers and non-fallers is shown in Figure 6 using a line chart. To classify an older adult as a recurrent faller or non-faller, the feature vector, namely*, Gait_Pattern* (Eq. 5) was thus based on the gait stability and symmetry characteristics. Since 27 participants performed the gait experiments twice, 54 sets of experimental data were acquired for the purpose of developing the *Gait_Pattern* feature vectors. There were 24 vectors for recurrent fallers and 30 vectors for non-fallers, which could be inputted into the kNN algorithm for classification. The dataset and its features, including the values of mean and variability of left-to-left *NCC* series (LL), left-to-right *NCC* series (LR), right-to-right *NCC* series (RR), and right-to-left *NCC* series (RL), of the x-axis components and RSS values are shown in Supplementary Tables S1, S2, respectively. There are no missing data. During the classification tests using the kNN algorithm, a 5-fold cross-validation strategy was applied. The original data clustering effects in both cases showed K = 3 to be the best choice for kNN (Figure 7) because K = 1 may result in a larger error due to overfitting. Therefore, we discarded the instance of K = 1 in this study. Table 2 showed the classification results for K = 3 based on the RSS and x-axis component of the 3D acceleration signals. The confusion matrix obtained by the kNN classifier was shown in Supplementary Table S3.

**TABLE 2:** Comparison of the kNN classification using normalized cross-correlation of the *x*-axis component and root-sum-of-square of the 3D acceleration signals.

Performance measures	Non-fallers	Recurrent fallers	Non-fallers	Recurrent fallers
	x-kNN		RSS-kNN	
Sensitivity (%)	93	88	97	92
Specificity (%)	88	93	92	97
Precision (%)	90	91	94	96
Recall (%)	93	88	97	92
F1-score (%)	92	89	95	94
Overall accuracy (%)	91		94	
MCC	0.812		0.887	

3D, three-dimensional; kNN, k-nearest neighbor; x-kNN, the kNN classification using the x-axis components; RSS-kNN, the kNN, classification using the RSS values; MCC, matthews correlation coefficient.

We also compared the efficiency of the kNN classification using the x-axis components (abbreviated as x-kNN) and RSS values (abbreviated as RSS-kNN). It is obvious that the classification results of RSS-kNN were superior to those of x-kNN, as shown in Tables 2. With x-kNN, the classification accuracy was 0.91 (91%) and Matthews correlation coefficient (MCC) (Chicco and Jurman, 2020) was 0.812 (Table 2). With RSS-kNN, the accuracy and MCC were even higher, at 0.94 (94%) and 0.887, respectively (Table 2). The classification advantage might have been due to the sensor’s calibration issue mentioned previously, such that various classification errors were produced by x-kNN for classifying non-fallers to recurrent fallers, as listed in Table 2.

## Discussion

**FIGURE 8:**
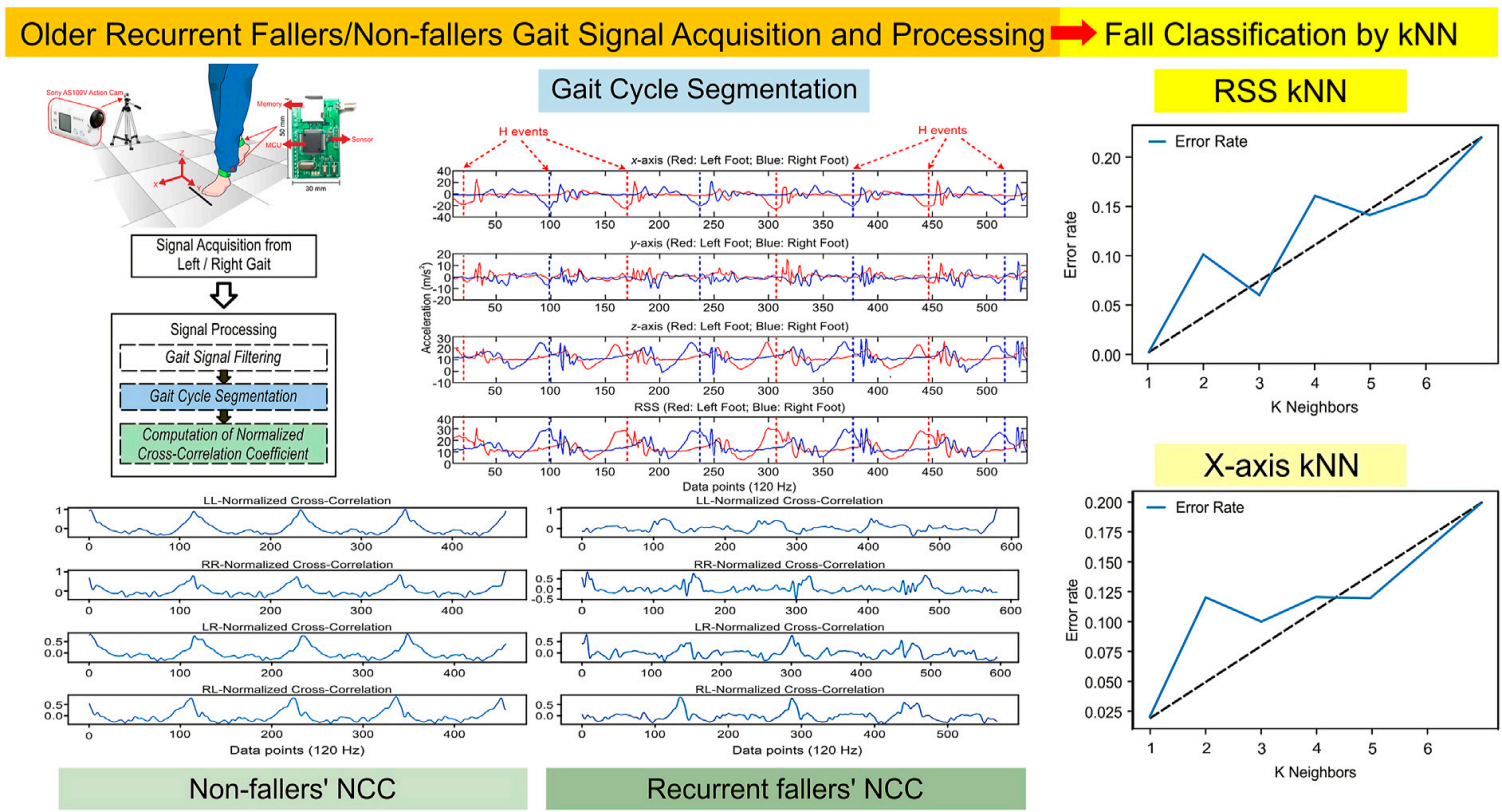
Summary illustration of this study.

In this study, we combined gait symmetry and stability with kNNs and obtained good classification results that distinguished between non-fallers and recurrent fallers. Our results, combined with those of recent studies, strengthen the role of gait stability and symmetry in the detection of older adult risks of falling, where the application of RSS-kNN was shown to be effective (Figure 8). Herein, kNN with an NCC analysis of gait signals was shown to classify older non-fallers and recurrent fallers effectively compared with the findings of previous studies (Drover et al., 2017; Hua et al., 2018; Tunca et al., 2020; Wu et al., 2020). In these related studies, IMU-based machine learning algorithms were adopted, such as random forest, kNN, a support vector machine, and long short-term memory, with accuracies ranging from 0.77 to 0.92. To our knowledge, this study is the first to use an NCC analysis and kNN to develop a fall-risk assessment model among older adults living in the community. With x-kNN and RSS-kNN, the classification accuracies were 0.91 and 0.94, respectively, and the MCCs were 0.812 and 0.887, respectively (Table 2). The classification results using RSS-kNN in this study were better than those obtained in previous studies. According to Gillain et al. (2019) and Commandeur et al. (2018) gait symmetry and stability are related to a risk of falling, but no single parameter was found that could represent for overall fall risk (Haescher et al., 2020). In this study, we combined gait symmetry and stability with kNNs and obtained good classification results that distinguished between non-fallers and recurrent fallers.

We observed four crucial points related to assessing the risk of falls in community-dwelling older adults. First, the properties of gait stability and symmetry can reveal natural differences in fall risk. In an older adult, a stable unilateral gait and a consistent gait between the left and right sides is important to balance the sensorimotor gait strategy required for walking safely. Second, the inertial sensors calibration issue is rather important in fall risk assessment for older adults. From the observations shown in Figure 7, the error rate of the K value selection for the IMU gait data sources in the x-axis component of the 3D acceleration signals was higher than that for the RSS. Because the RSS values are directionless, their magnitudes are stationary. However, owing to the gait motion during walking and direction in which the sensor is installed on the leg-sensing point, the magnitude of the x-axis component is not fixed and may vary substantially. Therefore, the use of the RSS value in fall risk assessment is paramount. Third, significant signal magnitude variations disrupt signal classification. Through NCC processing, the acceleration gait signals can be converted to a range from −1 to +1, such that an *NCC* series of gait stability and symmetry can be generated through the procedure proposed above. Thus, signal magnitude disturbance can be greatly reduced. Finally, in Tables 2, the accuracy of the fall risk assessment in RSS-kNN ( = 0.94) or x-kNN ( = 0.91) was found to be suitable for clinical settings for either screening or follow-up purposes. Especially in RSS-kNN, the classification accuracy reveals that by using the feature vector *Gait_Pattern*, the *NCC* series for recurrent fallers or non-fallers can be naturally clustered, such that they can be successfully distinguished. In addition, the kNN algorithms were based on clinically relevant fall history data; therefore, the algorithmic evaluation is a great advantage in clinical decision-making of fall-risk evaluation in geriatric care.

We also found that k = 3 was the optimal k. This was not computationally intensive and thus was suitable for a clinical setting for screening or follow-up. The *NCC* between the accelerometer signals of the bilateral ankles has been suggested to be an acceptable index of gait symmetry in stroke patients (Lien et al., 2017). The *NCC* between the accelerometer signals of the serial gait cycles in the unilateral ankle was representative of the gait signal stability. In this study, we normalized each gait cycle to 100% to control for temporal variabilities, where the kNN classification revealed different gait characteristics in non-fallers and recurrent fallers. The combination of *NCC* using the x-axis and RSS components in the bilateral and unilateral lower extremities in the kNN algorithm was accurate in the fall-risk assessment model. This indicates that both gait symmetry and stability are important components in determining fall risk in older adults. Previous studies have also reported that increased temporal gait variability reflects an unstable gait with a lack of the ability to exert a similar muscle force in repetitive gait cycles in older adults (Herman et al., 2005; Kwon et al., 2018).

The human gait, as is the case with other repetitive movements, typically consists of closed and open kinematic chains that commonly interchange during lower limb movement. In the swing phase, the limbs follow an open kinematic chain such that the velocity of the distal part correlates with that of the proximal part. Additionally, the deceleration of the proximal parts is associated with the inertia acceleration in the distal parts (McMullen and Uhl, 2000). In the stance phase (the closed kinetic chain), the stability of the proximal parts is based on the stability provided by the distal parts. In this study, the stability and symmetry transitions of the ankle and foot were less smooth in recurrent fallers (Figure 5), this may have affected the ability of the subject to stabilize the torso, along with proprioception from fixed body segments (Aminian et al., 1999). Recent studies investigating gait training using a lower limb robotic exoskeleton found that, after gait training, the ratio of gait cycle crossing zero moment point is reduced, and the gait phase transitions became more stable than those before training (Deng et al., 2019). Further studies exploring robotic exoskeleton gait training (Deng et al., 2019) intended to improve the transition between gait stability and gait symmetry and to restore normal gait phase transitions, especially among individuals with a recurrent fall history, are warranted.

There are limitations in this study that should be acknowledged. First, the participants were recruited from only two community service facilities in southern Taiwan. Although the results of the kNN classification were accurate, the limited number of facilities was insufficient to generalize the conclusion drawn from this study to all older adults. Further studies enrolling more community service facilities and a larger sample of older adults are needed to improve the generalizability and granularity of the kNN algorithm. Second, this study had a cross-sectional design; consequently, we could not claim a causal effect between gait stability and symmetry on fall events. In other words, it is still uncertain whether the impaired gait symmetry or stability of older fallers contributes to falls, or if recurrent fall events alter gait characteristics. Thus, further longitudinal research is encouraged for the classification algorithm of gait assessment based on fall history to enhance our understanding of the relationship between gait symmetry and stability and fall risk in older populations living in the community. Finally, the wearable sensors used in this study did not include gyroscopes as a directional measurement or surface electromyography as a muscle activation measurement; therefore, further studies with wearable sensors combining accelerometers, gyroscopes, and others are recommended to maximize the IMU functions.

## Conclusion

The study findings indicate that this novel assessment method using an instrumental gait analysis can be included in the routine clinical evaluation of gait patterns in older adults and that the RSS-based *NCC* coefficients can serve as effective gait features to assess the risk of falls, suggesting that both gait symmetry and stability are important components in determining the risk of falls in older adults. Interventions or rehabilitation therapies focused on improving gait patterns are important to reduce the incidence of falls among community-dwelling older adults.

## Data Availability

The original contributions presented in the study are included in the article/Supplementary Material, further inquiries can be directed to the corresponding authors.

## Abbreviations

10MWT, 10-m walk test; 3D, three-dimensional; BMI, body mass index; IMU, inertial measurement unit; kNN, k-nearest neighbor; NCC, normalized cross-correlation; RSS, root sum of squares; MCC, Matthews correlation coefficient.
