# Supplementary material for: Intelligent Fall-Risk Assessment Based on Gait Stability and Symmetry Among Older Adults Using Tri-Axial Accelerometry
Source: Front Bioeng Biotechnol. 2022 May 13;10:887269. doi: 10.3389/fbioe.2022.887269 (PMC9136169; doi:10.3389/fbioe.2022.887269)
Supplement: Supplementary file 1 [file DataSheet1.docx]

***Supplementary Materials***

**Supplementary Methods**

***The performance of the k-nearest neighbor (kNN) algorithm***

The accuracy, sensitivity, specificity, precision, and F1 scores of the experiments were adopted to evaluate the performance of the kNN algorithm and normalized cross-correlation analysis in classifying fallers and non-fallers, letting TP, FP, TN, and FN be the sets of true positive (correctly identified), false positive (incorrectly identified), true negative (correctly rejected), and false negative (incorrectly rejected) cases, respectively. The formulae for accuracy (Eq. (1)), sensitivity (Eq. (2)), specificity (Eq. (3)), precision (Eq. (4)), recall (Eq. (5)), F1 scores (Eq. (6)), and Matthews correlation coefficient (MCC) (Chicco and Jurman, 2020) (Eq. (7)) are expressed as follows:

$Accuracy=\frac{\left| TP \right|+\left| TN \right|}{\left| TP \right|+\left| TN \right|+\left| FP \right|+\left| FN \right|}$ , (1)

$Sensitivity=\frac{\left| TP \right|}{\left| TP \right|+\left| FN \right|}$ , (2)

$Specificity=\frac{\left| TN \right|}{\left| TN \right|+\left| FP \right|}$ (3)

$Precision=\frac{\left| TP \right|}{\left| TP \right|+\left| FP \right|}$ , (4)

$Recall=\frac{\left| TP \right|}{\left| TP \right|+\left| FN \right|}$ (5)

$F1 score=\frac{2*\left| TP \right|}{2*\left| TP \right|+\left| FP \right|+\left| FN \right|}$ , and (6)

$MCC=\frac{TP\cdot TN-FP\cdot FN}{\sqrt{\left( TP+FP \right)\cdot\left( TP+FN \right)\cdot\left( TN+FP \right)\cdot\left( TN+FN \right)}}$ (7)

where |•| is the cardinality of a set.

**Reference**

Chicco, D., and Jurman, G. (2020). The advantages of the Matthews correlation coefficient (MCC) over F1 Score and accuracy in binary classification evaluation. BMC Genomics. 21, 6. doi: 10.1186/s12864-019-6413-7.

**Supplementary Tables**

**Supplementary Table 1**. The raw data of mean and variability of left-to-left *NCC* series (LL), left-to-right *NCC* series (LR), right-to-right *NCC* series (RR), and right-to-left *NCC* series (RL) for recurrent fallers (Rec-F) and non-fallers (Non-F) of the x-axis components of the 3D acceleration signals.

|  | RR |  | LL |  | RL |  | LR |  |
| --- | --- | --- | --- | --- | --- | --- | --- | --- |
| Participants | mean | variability | mean | variability | mean | variability | mean | variability |
| Rec-F_01 | 0.0028 | 0.03926 | 0.00284 | -0.01151 | 0.0024 | 0.03964 | 0.00195 | 0.03944 |
|  | -0.0036 | -0.00233 | -0.00267 | 0.00042 | -0.01335 | -0.00604 | 0.00772 | 0.00322 |
| Rec-F_02 | -0.00116 | -0.01408 | -0.01026 | 0.01684 | -0.00008 | -0.00307 | -0.00951 | -0.0024 |
|  | -0.00106 | 0.04053 | 0.00485 | 0.00228 | -0.00029 | 0.01621 | 0.00496 | 0.01661 |
| Rec-F_03 | 0.01172 | -0.00446 | 0.00086 | -0.01271 | 0.0107 | -0.00792 | 0.00112 | -0.01295 |
|  | 0.01839 | 0.00705 | -0.00799 | 0.00369 | 0.01813 | 0.01153 | -0.00788 | -0.00307 |
| Rec-F_04 | -0.00086 | -0.01735 | 0.0001 | 0.03854 | -0.00051 | 0.03994 | -0.00019 | 0.03907 |
|  | 0.00188 | 0.01181 | -0.0101 | 0.04884 | 0.00943 | 0.03856 | -0.01077 | 0.01987 |
| Rec-F_05 | 0.01342 | 0.03302 | 0.01178 | -0.00023 | 0.01175 | 0.00813 | 0.01321 | 0.02043 |
|  | 0.0067 | 0.01971 | 0.00065 | 0.02926 | 0.0072 | 0.0227 | 0.0008 | 0.02152 |
| Rec-F_06 | 0.00134 | 0.01327 | 0.00158 | -0.00343 | -0.00612 | -0.00321 | 0.00514 | -0.00248 |
|  | 0.00067 | 0.01895 | -0.00414 | 0.03062 | -0.00928 | 0.02429 | -0.00089 | 0.02161 |
| Rec-F_07 | -0.00247 | 0.03605 | 0.0028 | 0.02486 | -0.00241 | 0.02748 | 0.00244 | 0.02709 |
|  | -0.00305 | 0.00722 | -0.00417 | -0.01027 | -0.00254 | -0.00329 | -0.00496 | -0.00149 |
| Rec-F_08 | 0.00848 | 0.03895 | -0.00091 | 0.00825 | 0.00749 | -0.00309 | -0.00148 | -0.01342 |
|  | -0.00216 | 0.00024 | 0.00307 | 0.02702 | 0.00048 | 0.03498 | 0.00032 | 0.0378 |
| Rec-F_09 | -0.00422 | 0.00088 | -0.01129 | 0.03257 | -0.0051 | -0.01625 | -0.02167 | 0.03346 |
|  | -0.00224 | 0.03423 | 0.01114 | 0.00183 | -0.00407 | -0.01407 | 0.00888 | -0.01701 |
| Rec-F_10 | -0.0004 | 0.03619 | -0.00782 | -0.01566 | -0.00088 | -0.00677 | -0.01214 | 0.00752 |
|  | -0.00331 | -0.00364 | -0.0001 | -0.01515 | -0.00299 | -0.00893 | 0.00016 | -0.01279 |
| Rec-F_11 | 0.05562 | 0.03839 | -0.00458 | 0.03579 | 0.04318 | 0.0362 | -0.00144 | 0.02989 |
|  | 0.0053 | 0.03872 | 0.00514 | -0.0122 | 0.00539 | -0.01828 | 0.00357 | -0.01884 |
| Rec-F_12 | -0.00526 | 0.03958 | 0.00089 | 0.02476 | 0.00328 | 0.03607 | -0.00478 | 0.02851 |
|  | -0.00103 | 0.01522 | -0.00337 | 0.02161 | -0.00701 | 0.01449 | 0.00147 | 0.01877 |
| Non-F_01 | 0.00074 | 0.06802 | 0.00596 | 0.06701 | 0.00067 | 0.06526 | 0.00571 | 0.06819 |
|  | 0.00471 | 0.04661 | 0.0056 | 0.06145 | 0.00442 | 0.04842 | 0.00532 | 0.04939 |
| Non-F_02 | 0.00363 | 0.06869 | 0.00629 | 0.05105 | 0.00336 | 0.05453 | 0.00607 | 0.05581 |
|  | 0.00306 | 0.06994 | 0.00202 | 0.05853 | 0.00291 | 0.06366 | 0.00186 | 0.06174 |
| Non-F_03 | 0.00296 | 0.07386 | 0.00585 | 0.06387 | 0.00281 | 0.06105 | 0.00509 | 0.06244 |
|  | 0.00137 | 0.07542 | 0.00288 | 0.05907 | 0.00147 | 0.06282 | 0.00266 | 0.06317 |
| Non-F_04 | 0.00541 | 0.06872 | 0.00525 | 0.04884 | 0.00527 | 0.05253 | 0.00513 | 0.05243 |
|  | 0.00229 | 0.05384 | 0.00453 | 0.04074 | 0.0023 | 0.04353 | 0.00414 | 0.04448 |
| Non-F_05 | 0.00757 | 0.05267 | 0.00446 | 0.03953 | 0.00682 | 0.04174 | 0.00446 | 0.0425 |
|  | -0.00218 | 0.07591 | 0.00091 | 0.08633 | 0.00089 | 0.08222 | -0.00225 | 0.0761 |
| Non-F_06 | 0.00366 | 0.06597 | 0.00208 | 0.05067 | 0.0036 | 0.05551 | 0.00202 | 0.05581 |
|  | 0.00047 | 0.06552 | -0.00596 | 0.04807 | 0.00548 | 0.05829 | -0.00902 | 0.05187 |
| Non-F_07 | 0.00317 | 0.07215 | 0.00063 | 0.0932 | 0.00286 | 0.0793 | 0.00103 | 0.08041 |
|  | 0.00297 | 0.10498 | 0.00507 | 0.08761 | 0.00301 | 0.09102 | 0.00471 | 0.0924 |
| Non-F_08 | 0.00275 | 0.09802 | 0.00501 | 0.08559 | 0.00246 | 0.08836 | 0.00477 | 0.09055 |
|  | -0.00286 | 0.064 | -0.00294 | 0.07295 | -0.01172 | 0.05896 | 0.00419 | 0.07398 |
| Non-F_09 | -0.00505 | 0.08688 | -0.00658 | 0.05213 | -0.00813 | 0.06192 | -0.00469 | 0.06762 |
|  | -0.00097 | 0.04554 | -0.00388 | 0.0578 | 0.00039 | 0.04708 | -0.00746 | 0.04571 |
| Non-F_10 | -0.00125 | 0.04213 | -0.00924 | 0.05429 | -0.00348 | 0.04730 | -0.00834 | 0.04701 |
|  | -0.00278 | 0.03476 | 0.00252 | 0.0596 | -0.0039 | 0.0438 | 0.00316 | 0.04252 |
| Non-F_11 | -0.01098 | 0.07248 | -0.01038 | 0.0547 | -0.00969 | 0.05952 | -0.01091 | 0.06229 |
|  | -0.00806 | 0.06929 | -0.00231 | 0.06705 | -0.00672 | 0.06689 | -0.0035 | 0.06685 |
| Non-F_12 | -0.0096 | 0.05532 | 0.00112 | 0.05365 | -0.0049 | 0.05679 | -0.00272 | 0.04744 |
|  | 0.00674 | 0.05795 | 0.00196 | 0.0605 | 0.00745 | 0.062 | 0.0014 | 0.0525 |
| Non-F_13 | -0.00453 | 0.04336 | 0.00407 | 0.05863 | -0.00511 | 0.04858 | 0.00348 | 0.04658 |
|  | -0.00846 | 0.05408 | -0.00871 | 0.04362 | 0.00369 | 0.04862 | -0.01643 | 0.04813 |
| Non-F_14 | -0.00284 | 0.02052 | -0.00243 | 0.04185 | -0.00623 | 0.02878 | -0.00097 | 0.02402 |
|  | -0.00186 | 0.01719 | -0.00081 | 0.01588 | -0.00131 | 0.01684 | -0.00307 | 0.0154 |
| Non-F_15 | -0.00847 | 0.03688 | -0.00763 | 0.04638 | -0.01251 | 0.04517 | -0.00582 | 0.03589 |
|  | -0.00334 | 0.0332 | -0.00661 | 0.03204 | -0.00671 | 0.02897 | -0.0025 | 0.03308 |

NCC: normalized cross-correlation; 3D: three-dimensional

**Supplementary Table 2**. The raw data of mean and variability of left-to-left *NCC* series (LL), left-to-right *NCC* series (LR), right-to-right *NCC* series (RR), and right-to-left *NCC* series (RL) for recurrent fallers (Rec-F) and non-fallers (Non-F) of the root-sum-of-square (RSS) values of the 3D acceleration signals.

|  | RR |  | LL |  | RL |  | LR |  |
| --- | --- | --- | --- | --- | --- | --- | --- | --- |
| Participants | mean | variability | mean | variability | mean | variability | mean | variability |
| Rec-F_01 | 0.00863 | 0.0647 | 0.00398 | 0.06507 | 0.00811 | 0.06759 | 0.00392 | 0.06098 |
|  | -0.00528 | 0.06533 | -0.00252 | 0.06679 | -0.01571 | 0.05166 | 0.00692 | 0.07062 |
| Rec-F_02 | 0.00031 | 0.06322 | -0.00992 | 0.06614 | -0.00279 | 0.07285 | -0.01246 | 0.05244 |
|  | 0.0037 | 0.07126 | 0.00575 | 0.09464 | 0.00469 | 0.0989 | 0.00532 | 0.09903 |
| Rec-F_03 | 0.00749 | 0.07991 | 0.00044 | 0.08407 | 0.0056 | 0.05123 | 0.00145 | 0.09296 |
|  | 0.02325 | 0.04613 | -0.00791 | 0.09409 | 0.02295 | 0.04774 | -0.00922 | 0.08703 |
| Rec-F_04 | 0.00032 | 0.04963 | 0.0013 | 0.08781 | -0.00053 | 0.09904 | 0.00188 | 0.09619 |
|  | -0.00184 | 0.07814 | -0.01664 | 0.06592 | 0.00818 | 0.05969 | -0.01438 | 0.0855 |
| Rec-F_05 | 0.01065 | 0.02459 | 0.00995 | 0.01729 | 0.00356 | 0.01635 | 0.01681 | 0.02767 |
|  | 0.00321 | 0.06544 | -0.00254 | 0.06604 | 0.00405 | 0.09861 | -0.0009 | 0.06755 |
| Rec-F_06 | -0.00667 | 0.06736 | 0.00291 | 0.05498 | -0.01717 | 0.04342 | 0.00221 | 0.07055 |
|  | -0.00595 | 0.02427 | -0.00424 | 0.01613 | -0.00882 | 0.01733 | -0.00057 | 0.02786 |
| Rec-F_07 | -0.00458 | 0.05267 | 0.00573 | 0.09663 | -0.00476 | 0.06669 | 0.0045 | 0.06936 |
|  | -0.00883 | 0.01197 | 0.0046 | 0.01728 | 0.0005 | 0.01617 | -0.00984 | 0.0126 |
| Rec-F_08 | 0.02952 | 0.04725 | 0.01718 | 0.04901 | 0.02419 | 0.04409 | 0.02154 | 0.05033 |
|  | -0.02258 | 0.03508 | -0.00156 | 0.01503 | -0.00212 | 0.01979 | -0.0239 | 0.02235 |
| Rec-F_09 | 0.00555 | 0.02386 | -0.00813 | 0.05173 | -0.00651 | 0.04217 | 0.00151 | 0.02996 |
|  | -0.01938 | 0.0439 | -0.01214 | 0.02334 | -0.01423 | 0.02731 | -0.01596 | 0.03546 |
| Rec-F_10 | -0.00779 | 0.0085 | -0.01067 | 0.01361 | -0.01366 | 0.01047 | -0.01097 | 0.00973 |
|  | -0.01614 | 0.01432 | -0.01097 | 0.01398 | -0.00928 | 0.01212 | -0.0195 | 0.01442 |
| Rec-F_11 | -0.00536 | 0.0789 | -0.01366 | 0.06397 | -0.00366 | 0.05601 | -0.01174 | 0.07102 |
|  | 0.00545 | 0.04359 | 0.00837 | 0.05807 | 0.00489 | 0.04849 | 0.00745 | 0.05012 |
| Rec-F_12 | -0.00786 | 0.05499 | -0.00239 | 0.06708 | -0.00257 | 0.06005 | -0.00918 | 0.06246 |
|  | -0.00898 | 0.0567 | 0.00255 | 0.04411 | -0.00964 | 0.04266 | 0.00739 | 0.04547 |
| Non-F_01 | 0.00031 | 0.19458 | 0.00834 | 0.20579 | 0.00027 | 0.19705 | 0.0079 | 0.20291 |
|  | 0.00603 | 0.19693 | 0.00702 | 0.1944 | 0.00558 | 0.1938 | 0.00694 | 0.19492 |
| Non-F_02 | 0.0012 | 0.19184 | 0.00292 | 0.20233 | 0.00107 | 0.19086 | 0.00297 | 0.19879 |
|  | 0.00115 | 0.18709 | -0.00066 | 0.20844 | 0.00173 | 0.19637 | -0.00119 | 0.19617 |
| Non-F_03 | 0.0017 | 0.20416 | 0.00551 | 0.18501 | 0.00118 | 0.1913 | 0.00512 | 0.19319 |
|  | 0.00338 | 0.19821 | 0.00239 | 0.18866 | 0.00315 | 0.19347 | 0.00217 | 0.19144 |
| Non-F_04 | 0.00279 | 0.17623 | 0.00486 | 0.18865 | 0.00287 | 0.1803 | 0.00464 | 0.18275 |
|  | 0.00279 | 0.17511 | 0.00455 | 0.18088 | 0.00255 | 0.17715 | 0.00447 | 0.1774 |
| Non-F_05 | 0.00358 | 0.17256 | 0.00414 | 0.17994 | 0.00309 | 0.17741 | 0.004 | 0.17266 |
|  | -0.00781 | 0.1796 | 0.00079 | 0.18634 | -0.0019 | 0.18148 | -0.0047 | 0.18193 |
| Non-F_06 | 0.00264 | 0.1683 | 0.00262 | 0.18318 | 0.00289 | 0.17412 | 0.00227 | 0.17421 |
|  | -0.00174 | 0.15046 | -0.00781 | 0.20064 | 0.00436 | 0.17429 | -0.01148 | 0.16853 |
| Non-F_07 | 0.00298 | 0.15321 | 0.0046 | 0.15673 | 0.00273 | 0.15303 | 0.00455 | 0.15423 |
|  | 0.00465 | 0.15653 | 0.00491 | 0.15087 | 0.00467 | 0.15049 | 0.00484 | 0.1527 |
| Non-F_08 | 0.00444 | 0.15163 | 0.00707 | 0.1535 | 0.00442 | 0.14894 | 0.00688 | 0.15263 |
|  | -0.00311 | 0.17612 | -0.00136 | 0.13379 | -0.00695 | 0.12882 | 0.00376 | 0.18208 |
| Non-F_09 | -0.00284 | 0.14871 | -0.00263 | 0.15831 | -0.00674 | 0.14632 | 0.002 | 0.15975 |
|  | -0.00393 | 0.15025 | -0.00811 | 0.16053 | -0.00247 | 0.1534 | -0.0096 | 0.15567 |
| Non-F_10 | -0.00861 | 0.17194 | -0.01505 | 0.12067 | -0.01298 | 0.12867 | -0.01594 | 0.15682 |
|  | -0.00439 | 0.14871 | 0.00274 | 0.10326 | -0.00466 | 0.11701 | 0.00359 | 0.12153 |
| Non-F_11 | -0.01139 | 0.11638 | -0.01249 | 0.12786 | -0.0081 | 0.11363 | -0.01085 | 0.1234 |
|  | -0.00759 | 0.10244 | -0.0087 | 0.12913 | -0.00402 | 0.1181 | -0.00921 | 0.11301 |
| Non-F_12 | -0.00394 | 0.12152 | -0.00404 | 0.09907 | -0.00111 | 0.10897 | -0.005 | 0.09629 |
|  | 0.01092 | 0.10041 | 0.00193 | 0.08777 | 0.01117 | 0.10144 | 0.00233 | 0.08452 |
| Non-F_13 | 0.00137 | 0.10056 | 0.00812 | 0.08649 | 0.00184 | 0.08733 | 0.00653 | 0.09434 |
|  | -0.01004 | 0.11079 | -0.01281 | 0.09929 | 0.00054 | 0.08823 | -0.01924 | 0.10497 |
| Non-F_14 | -0.00736 | 0.07533 | 0.00187 | 0.09628 | -0.0045 | 0.07733 | 0.00191 | 0.08995 |
|  | -0.00338 | 0.07637 | -0.01327 | 0.08403 | -0.00414 | 0.07858 | -0.01524 | 0.07507 |
| Non-F_15 | -0.01145 | 0.04805 | -0.01681 | 0.12839 | -0.01768 | 0.08485 | -0.01409 | 0.06205 |
|  | -0.0072 | 0.06129 | -0.01448 | 0.09113 | -0.01384 | 0.06348 | -0.00756 | 0.06516 |

NCC: normalized cross-correlation; 3D: three-dimensional

**Supplementary Table 3**. Confusion matrix of the kNN classification using normalized cross-correlation of the x-axis components and root-sum-of-square (RSS) values of the 3D acceleration signals.

|  | x-axis component | | RSS | |
| --- | --- | --- | --- | --- |
|  | Predicted class | | Predicted class | |
|  | Non-faller | Recurrent faller | Non-faller | Recurrent faller |
| Non-faller | 93.3% | 6.7% | 96.7% | 3.3% |
| Recurrent faller | 12.5% | 87.5% | 8.3% | 91.7% |

kNN: k-nearest neighbor; RSS: root-sum-of-square; 3D: three-dimensional
